# High frequency audible calls in northern birch mice *Sicista betulina* in response to handling: effects of individuality, sex and body mass on the acoustics

**DOI:** 10.1186/s13104-019-4719-9

**Published:** 2019-10-22

**Authors:** Ilya A. Volodin, Anna V. Klenova, Olga G. Ilchenko, Elena V. Volodina

**Affiliations:** 1 Department of Vertebrate Zoology, Faculty of Biology, Lomonosov Moscow State University, Vorobievy Gory, 1/12, Moscow, 119234 Russia; 2 Scientific Research Department, Moscow Zoo, B. Gruzinskaya, 1, Moscow, 123242 Russia

**Keywords:** Rodent, Distress calls, Acoustic communication, Ultrasonic vocalization, Emotional arousal, Birch mouse, Individuality, Body size, Sexual dimorphism, Dipodoidea

## Abstract

**Objectives:**

This is the first study of the sonic and ultrasonic vocalization in a Dipodidae rodent. For the small-sized quadrupedal northern birch mouse *Sicista betulina*, phylogenetically related to the bipedal jerboas (Dipodidae), we report null results for ultrasonic vocalization and investigate the acoustic cues to individual identity, sex and body size in the discomfort-related high-frequency tonal sonic calls.

**Results:**

We used a parallel audio recording in the sonic and ultrasonic ranges during weighting adult northern birch mice before the scheduled hibernation in captivity. The sonic (audible) high-frequency tonal calls (ranging from 6.21 to 9.86 kHz) were presented in all individuals (7 males and 4 females). The ultrasonic calls lacked in the recordings. Two-way nested ANOVA revealed the effects of caller individual identity on all 10 measured acoustic variables and the effects of sex on four out of 10 measured acoustic variables. Discriminant function analyses with 10 acoustic variables included in the analysis showed 85.5% correct assignment of calls to individual and 79.7% correct assignment of calls to sex; both values significantly exceeded the random values (23.1% and 54.3%, respectively) calculated with randomization procedure. Body mass did not differ between sexes and did not correlate significantly with the acoustic variables.

## Introduction

Adult rodents may vocalize during handling in the lab [1–5]. These calls probably have no special function, being triggered by the elevated emotional arousal and discomfort of a caller [3, 4, 6–9]. Nevertheless, these calls may provide information about the caller, presented in the mammalian calls by default. Mammalian calls are offprints of individual vocal apparatus of a caller [10] and therefore by default provide information about caller’s individual identity at level higher than by chance [11–17]. Call variables may provide general information about body size [18, 19] and particular information about body mass [20] and body condition [21–24]. In addition, acoustic traits may reflect sexual dimorphism [14, 25–28].

Small mammals with their respectively small sound-producing structures commonly produce high-frequency sonic calls [29–31] along with ultrasonic calls above 20 kHz [32, 33]. Birch mice (genus *Sicista,* family Smithidae) are quadrupedal rodents, related to the bipedal jerboas Dipodidae and comprising together superfamily Dipodoidea. The northern birch mouse *Sicista betulina*, which status as a separate species was recently confirmed based on cytochrome *b* gene polymorphism [34], inhabit temperate forests and taiga from Western Europe to the Baikal region. This is a small mammal, with female body mass 9.4 ± 0.22 g and male body mass 8.98 ± 0.06 [35], maximum 10.4 g [36].

Northern birch mice are nocturnal but have bursts of activity during the day in spring and autumn [37] and hibernate for more than 7 months of the year [38]. There are no sustainable laboratory colonies of this species, as these animals poorly live in captivity, only for some months [37, 38]. At Moscow Zoo (Moscow, Russia), a temporal captive colony of northern birch mice emerged in 2018 from animals saved by volunteers during mass migration at peak of population growth, when many individuals were perishing in river water.

Calls of any Dipodoidea species have never been previously investigated. Zootechnical routine before the scheduled seasonal hibernation of small mammals at Moscow Zoo includes obligatory visual inspection and weighing of all individuals to estimate their body condition. Preliminary observations of the authors indicated that birch mice vocalize in human audible frequency range during this procedure. We expected that at handling, these small-sized rodents would vocalize in both the sonic and ultrasonic ranges, as e.g. laboratory rats [2] or some species of gerbils [4, 5]. In this study, we apply audio recording in both sonic and ultrasonic ranges of frequencies to record the discomfort-related calls of the captive northern birch mice. We describe the acoustic structure and estimate the effects of individuality, sex and body mass on the acoustic variables of these calls.

## Main text

### Methods

Calls of 11 adult northern bitch mice (7 males, 4 females) were recorded from 12 to 17 September 2018 from members of a newly established captive colony of this species at Moscow Zoo (Moscow, Russia). All subjects were wild-captured in August 2018 on the eastern shore of the Yenisei River (Siberia, Russia) near the village Mirnoye (62°18′N 89°01′E).

The animals were kept under a natural light regime at temperature around 20 °C, singly in wire-and-plastic cages of 40 × 30 × 30 cm, with a bedding of mulch, soil, sand and enrichment of various shelters. They received custom-made small rodent chow with insect and mineral supplements and water ad libitum.

Calls of each animal were recorded during handling in daytime at temperature about 20 °C. Parallel 1–2-min recording in the sonic (20 Hz–20 kHz) and ultrasonic (over 20 kHz) ranges of frequencies was conducted during the 1–2-min inspection-and-weighing procedure for preparing the animal to the scheduled hibernation. During the procedure, the animal was taken out of the home cage with a keeper hand, inspected visually, weighed on the electronic scales G&G TS-100 (G&G GmbH, Neuss, Germany) with 0.01 g precision and returned back to the cage. During recording, a researcher could always clearly see that calls were emitted by a focal animal. Animal disturbance was kept at minimum; no special actions provoking vocalization was applied.

For the sonic recording (sampling rate 48 kHz, 16 bit resolution) we used a Marantz PMD-660 solid state recorder (D&M Professional, Kanagawa, Japan) with Sennheiser K6-ME64 microphone (Sennheiser electronic, Wedemark, Germany), hand-held at distance 0.5–1 m from the animal. For the ultrasonic recordings (254 kHz, 16 bit resolution), we used an Echo Meter Touch 2Pro (Wildlife Acoustics Inc., Maynard, MA, USA) run at Android smartphone OnePlus 3 (OnePlus Company, BBK Electronics LTD, Shenzhen, Guangdong, China), hand-held at distance 0.5–1 m from the animal.

Each recording trial provided two simultaneously recorded wav-files of the same length per individual, one sonic and one ultrasonic. In total, 17 recording trials (one trial per individual for five subjects and two trials per individual separated with time spans of 2–5 days for six subjects), provided in total 34 (17 sonic and 17 ultrasonic) wav-files for spectrographic analysis. For subjects with two trials per individual, weighting data were averaged for analyses.

**Fig. 1:**
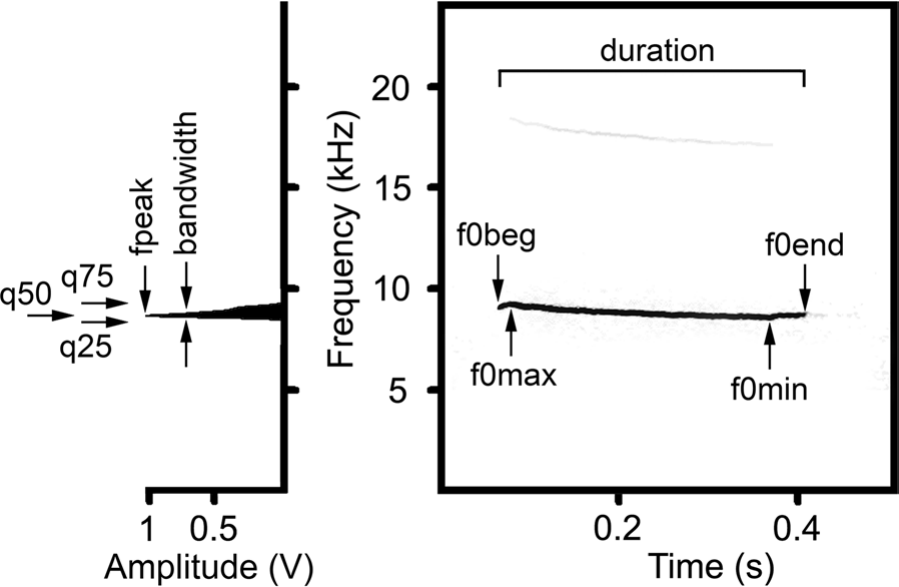
Measured acoustic variables in the discomfort sonic calls of the northern birch mice *Sicista betulina*. Spectrogram (right) and power spectrum (left). Designations: duration—call duration; f0_max_—the maximum fundamental frequency; f0_min_—the minimum fundamental frequency; f0_beg_—the start fundamental frequency; f0_end_—the end fundamental frequency; f_peak_—the frequency of maximum amplitude; q_25_, q_50_, q_75_—lower, medium and upper quartiles; bandwidth—the bandwidth of the f_peak_ at the distance of 10 dB from the maximum. The spectrogram was created with Hamming window; 48 kHz sampling rate; FFT 1024 points; frame 50%; and overlap 93.75%

Visual inspection of spectrograms of the wav-files using Avisoft SASLab Pro software (Avisoft Bioacoustics, Berlin, Germany) showed null results for presence of the ultrasonic calls, whereas all the sonic wav-files contained the same type of tonal high-frequency sonic calls. For acoustic analyses, we selected up to 20 (14–20) calls with good noise-to-call ratio per individual, 207 calls in total (Additional file 1: Table S1). If two recordings per animal were available, we selected calls in a balanced manner from both recordings. In each call, we measured 10 acoustic variables (Fig. 1). We measured, in the spectrogram window of Avisoft (sampling frequency 48 kHz, Hamming window, FFT 1024 points, frame 50%, overlap 96.87%, providing frequency resolution 47 Hz and time resolution 0.67 ms), call duration with the standard marker cursor, and the maximum fundamental frequency (f0_max_), the minimum fundamental frequency (f0_min_), the start fundamental frequency (f0_beg_) and the end fundamental frequency (f0_end_) with the reticule cursor. In each call, we also measured, in the power spectrum window of Avisoft, the frequency of maximum amplitude (f_peak_), the three quartiles (q_25_, q_50_ and q_75_) covering, respectively 25%, 50% and 75% of call energy from the call’s mean power spectrum, and the bandwidth of the f_peak_ at the distance of 10 dB from the maximum (Fig. 1). All measurements were exported to Microsoft Excel (Microsoft Corp., Redmond, WA, USA).

Statistical analyses were made with STATISTICA, v. 8.0 (StatSoft, Tulsa, OK, USA); all means are given as mean ± SD, and differences were considered significant whenever *p *< 0.05. Only three of the 110 distributions departed from normality (Kolmogorov–Smirnov test, *p* > 0.05). We used two-way nested ANOVA with individual nested within sex, sex as fixed factor and individual as random factor, to estimate the effects of individuality and sex on the acoustic variables. We used one-way ANOVA to compare body mass between sexes. We used Pearson correlation with logarithm of body mass as proxy of body size to estimate effects of body size on the acoustic variables. We used discriminant function analysis (DFA) standard procedure to estimate potential for encoding individuality and sex by the high-frequency sonic calls of the northern birch mice. We used all 10 acoustic variables because they weakly correlated to each other on the basis of cross-correlation analysis, thus meeting the assumptions of DFA. The relative contribution of each acoustic variable in the correct assignment of calls to individual was estimated based on Wilks’ Lambda values, the smaller is the value, the greater is the contribution of the given acoustic variable to the overall discrimination [39].

Random values (of correct assignment to individual or to sex by chance) were calculated using randomization procedure [40] in R (https://www.r-project.org). The random values were averaged from DFAs performed on 1000 randomized permutations on the data sets, as in [39, 41].

### Results

**Fig. 2:**
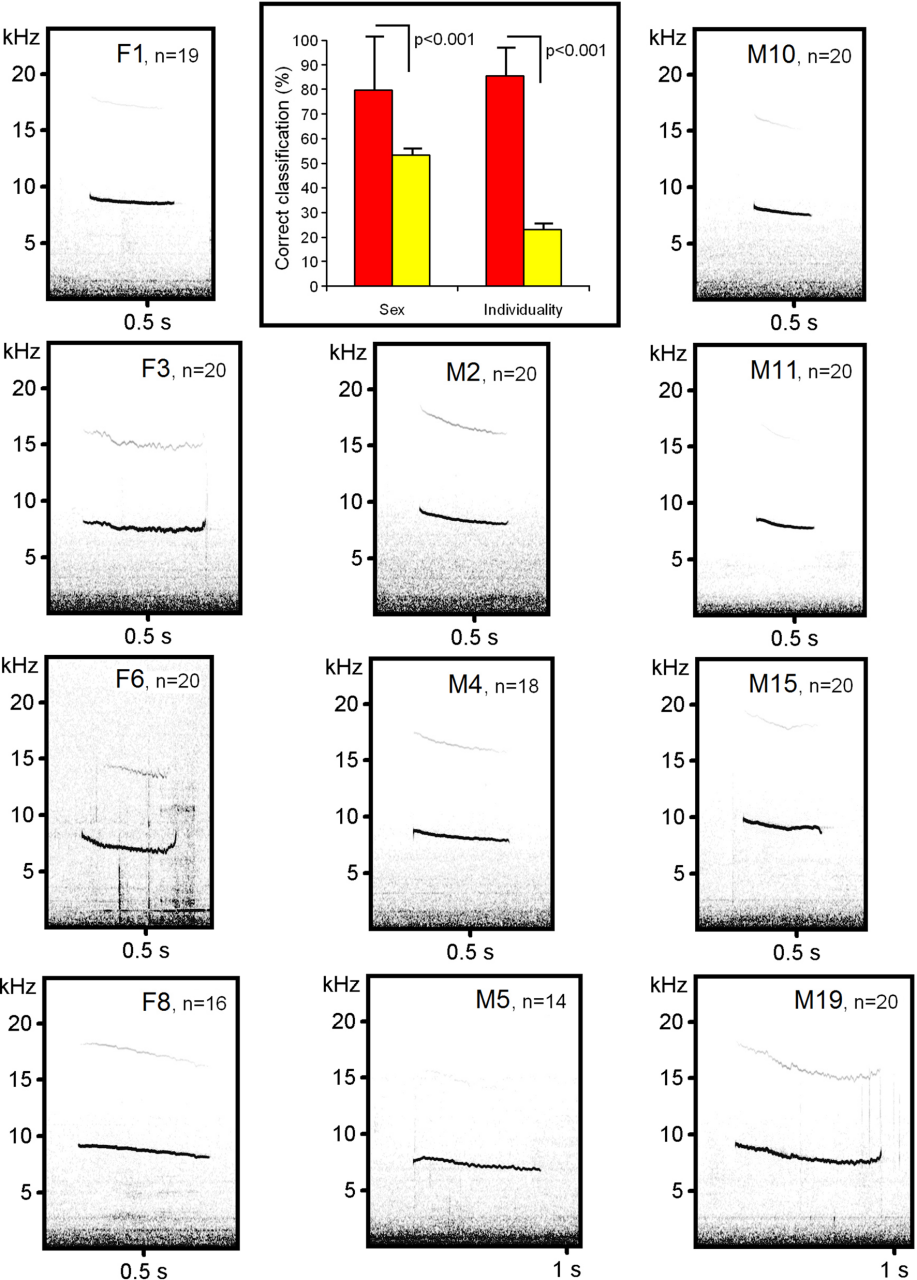
Individual and sexual identity in the discomfort sonic calls of the northern birch mice. Spectrograms of one call per individual are given for the 4 female (F1–F6) and 7 male (M2–M19) subject northern birch mice, n indicates the number of calls. Red bars represent the actual values of correct classifying of the discomfort calls to sex and to individual with DFA. Yellow bars represent the random (chance) values of correct classifying of the discomfort calls to sex and to individual with DFA. Comparisons between the actual and random values (indicated with brackets above the bars) were done using the permutation test. Bars indicate averages, whiskers indicate SD. The spectrogram was created with Hamming window; 48 kHz sampling rate; FFT 1024 points; frame 50%; and overlap 87.5%. Original wav-files are available in Additional file 2

We did not find the ultrasonic calls in the studied northern birch mice during the handling procedures. The found sonic calls were rather long (from 0.24 to 0.65 s in different individuals) and high-frequency. Between individuals, the f0_min_ ranged from 6.21 to 8.69 kHz, the f0_max_ ranged from 8.06 to 9.86 kHz (Table 1, Fig. 2, Additional files 1 and 2). A common pattern of frequency modulation was a steady decrease of frequency from call beginning to call end. The f0_max_ coincided with f0_beg_ in 96 of 207 (46.4%) calls, and was located in the first quarter of call duration in the other 91 (44.0%) calls. The f0_min_ coincided with f0_end_ in 80 (38.6%) calls, and was located in the last quarter of call duration in the other 92 (44.4%) calls. The values of f_peak_ were always in the range of the fundamental frequency band. The fundamental frequency band was the band with most energy in all calls without exclusion. In some individuals, the fundamental frequency increased again at the end of a call, what in 17 (8.2%) calls resulted in the coincidence of the f0_end_ and f0_max_ values (Fig. 2). Only four calls of one individual female contained nonlinear phenomena. Therefore, the high-frequency tonal calls of the northern birch mice had a very simple acoustic structure.

**Table 1:** Values of measured variables and their relationships with birch mouse sex, individuality and body mass

Variable	Mean ± SD value			ANOVA		Pearson correlation with log body mass
	All animal calls	Male calls	Female calls	Sex	Individual identity	
Duration (s)	0.46 ± 0.17	0.42 ± 0.17	0.54 ± 0.15	*F*_1,196_ = 39.7; *p *< 0.001	*F*_9,196_ = 22.8; p < 0.001	*r *= 0.29; *p *= 0.42
f0_max_ (kHz)	8.81 ± 0.61	8.83 ± 0.63	8.78 ± 0.57	*F*_1,196_ = 0.08; *p *= 0.78	*F*_9,196_ = 67.8; *p *< 0.001	*r *= 0.18; *p *= 0.62
f0_min_ (kHz)	7.61 ± 0.75	7.79 ± 0.55	7.31 ± 0.94	*F*_1,196_ = 65.6; *p *< 0.001	*F*_9,196_ = 82.5; *p *< 0.001	*r *= − 0.05; *p *= 0.90
f0_beg_ (kHz)	8.70 ± 0.71	8.70 ± 0.74	8.70 ± 0.65	*F*_1,196_ = 2.06; *p *= 0.15	*F*_9,196_ = 76.9; *p *< 0.001	*r *= 0.09; *p *= 0.80
f0_end_ (kHz)	7.95 ± 0.59	7.93 ± 0.57	7.99 ± 0.63	*F*_1,196_ = 2.93; *p *= 0.09	*F*_9,196_ = 29.3; *p *< 0.001	*r *= 0.28; *p *= 0.43
f_peak_ (kHz)	8.05 ± 0.74	8.14 ± 0.56	7.90 ± 0.98	*F*_1,196_ = 6.23; *p *= 0.01	*F*_9,196_ = 53.7; *p *< 0.001	*r *= − 0.16; *p *= 0.65
q_25_ (kHz)	7.92 ± 0.67	8.02 ± 0.56	7.75 ± 0.81	*F*_1,196_ = 16.3; *p *< 0.001	*F*_9,196_ = 68.3; *p *< 0.001	*r *= − 0.06; *p *= 0.87
q_50_ (kHz)	8.34 ± 0.68	8.31 ± 0.50	8.38 ± 0.93	*F*_1,196_ = 2.38; *p *= 0.12	*F*_9,196_ = 13.2; *p *< 0.001	*r *= − 0.01; *p *= 0.99
q_75_ (kHz)	9.40 ± 1.72	8.87 ± 0.65	10.33 ± 2.47	*F*_1,196_ = 73.1; *p *< 0.001	*F*_9,196_ = 24.8; *p *< 0.001	*r *= − 0.11; *p *= 0.75
Bandwidth (kHz)	0.73 ± 0.36	0.70 ± 0.34	0.77 ± 0.39	*F*_1,196_ = 2.06; *p *= 0.15	*F*_9,196_ = 6.67; *p *< 0.001	*r *= − 0.15; *p *= 0.67
Body mass (g)	12.63 ± 2.23	12.59 ± 2.21	12.74 ± 2.76	*F*_1,8_ = 0.01; *p *= 0.93		

Designations: duration—call duration; f0_max_—the maximum fundamental frequency; f0_min_—the minimum fundamental frequency; f0_beg_—the start fundamental frequency; f0_end_—the end fundamental frequency; f_peak_—the frequency of maximum amplitude; q_25_, q_50_, q_75_—lower, medium and upper quartiles; bandwidth—the bandwidth of the f_peak_ at the distance of 10 dB from the maximum; *p* estimates less than 0.05 are shown in underline

Two-way nested ANOVA revealed the effect of caller individual identity on all measured acoustic variables and the effect of sex on duration, f0_min_, f_peak_, q_25_ and q_75_ (Table 1). DFA showed 85.5% correct assignment of discomfort calls to individual, significantly exceeding the random value 23.1 ± 2.4% (permutation test, *p *< 0.001), and 79.7% correct assignment to sex, significantly exceeding the random value 54.3 ± 2.8% (permutation test, *p *< 0.001) (Fig. 2). The three acoustic variables that mainly contributed to discrimination to individual (in order of decreasing importance) were f0_beg_, f0_max_ and duration, and those that mainly contributed to discrimination to sex were f0_min_, f0_end_ and f_peak_. Body mass did not differ between sexes and did not significantly correlate with acoustic variables (Table 1).

### Discussion

This first study of vocalization in a Dipodidae rodent revealed that, in response to handling, adult northern birch mice of both sexes produced tonal calls of about 8–9 kHz (Fig. 2, Additional file 2). For presence of ultrasonic calls, we obtained negative results. The lack of ultrasonic calls was unexpected for such small mammal (lighter than 10 g [35] or about 12 g in this study, Table 1). Nevertheless, for some other small mammals, as shrews, convincing negative results also indicate the absence of ultrasonic calls [42, 43].

In the northern birch mice, we found the lack of sexual dimorphism in body size, corresponding to only a weak sexual dimorphism in the acoustic variables. At the same time, their calls of very simple acoustic structure provided strong cues to acoustic individuality. Similar data regarding the lack of sexual dimorphism in body size, similar acoustics between sexes and high potential of high-frequency tonal calls to encode caller’s individual identity in spite of their very simple acoustic structure, were obtained for the alarm calls of speckled ground squirrels *Spermophilus suslicus* [11, 13].

The study colony of Moscow Zoo consisted of wild-captured animals. Therefore, the collected acoustic material is valuable as reference data on vocalization, which was not yet affected by domestication, for comparison with data of further studies from colonies of birch mice in zoos and laboratories. In mammals kept in captivity for many generations, vocalization can be thoroughly changed compared to the founders [44, 45].

## Limitations

This pilot study had a few limitations:

- The study was conducted in one lab in one newly established population, on a limited number of individuals, what limits expansion of results for the entire species.
- Recordings were made within a short period of a few days and in one behavioural context (handling-related discomfort), therefore the detected absence of ultrasonic calls in this species requires confirmation for other behavioral contexts.
- Precise age of the study animals was unknown. They could be young, old, or individuals of different ages, so the potential age-related acoustic variation could affect vocal variables.

## Supplementary information

**Additional file 1: Table S1**. Data table with body mass and acoustic measurements of discomfort-related sonic calls of the study northern birch mice.

**Additional file 2: Audio S1.** Audio wav-file with discomfort-related sonic calls of 4 female and 7 male northern birch mice *Sicista betulina*, two calls per individual are given.

## Data Availability

The dataset supporting the conclusions of this article is included within the article and its additional files.

## Abbreviations

DFA: discriminant function analysis
f0_max_: the maximum fundamental frequency
f0_min_: the minimum fundamental frequency
f0_beg_: the start fundamental frequency
f0_end_: the end fundamental frequency
f_peak_: call maximum amplitude frequency
q_25_: call lower power quartile
q_50_: call medium power quartile
q_75_: call upper power quartile
